# The Small-Molecule Flunarizine in Spinal Muscular Atrophy Patient Fibroblasts Impacts on the Gemin Components of the SMN Complex and TDP43, an RNA-Binding Protein Relevant to Motor Neuron Diseases

**DOI:** 10.3389/fmolb.2020.00055

**Published:** 2020-04-17

**Authors:** Delphine Sapaly, Perrine Delers, Jennifer Coridon, Badih Salman, Franck Letourneur, Florent Dumont, Suzie Lefebvre

**Affiliations:** ^1^INSERM UMR-S 1124, Toxicité Environnementale, Cibles Thérapeutiques, Signalisation Cellulaire et Biomarqueurs, Campus Saint-Germain-des-Prés, Université de Paris, Paris, France; ^2^BioMedTech Facilities INSERM US36 - CNRS UMS 2009, Campus Saint-Germain-des-Prés, Université de Paris, Paris, France; ^3^Genom’ic Platform, INSERM U1016, Institut Cochin, Paris, France

**Keywords:** neurodegenerative disorders, spinal muscular atrophy, amyotrophic lateral sclerosis, RNA metabolism, nuclear bodies, SMN complex, Gemin, TDP-43

## Abstract

The motor neurodegenerative disease spinal muscular atrophy (SMA) is caused by alterations of the survival motor neuron 1 (SMN1) gene involved in RNA metabolism. Although the disease mechanisms are not completely elucidated, SMN protein deficiency leads to abnormal small nuclear ribonucleoproteins (snRNPs) assembly responsible for widespread splicing defects. SMN protein localizes in nuclear bodies that are lost in SMA and adult onset amyotrophic lateral sclerosis (ALS) patient cells harboring TDP-43 or FUS/TLS mutations. We previously reported that flunarizine recruits SMN into nuclear bodies and improves the phenotype of an SMA mouse model. However, the precise mode of action remains elusive. Here, a marked reduction of the integral components of the SMN complex is observed in severe SMA patient fibroblast cells. We show that flunarizine increases the protein levels of a subset of components of the SMN-Gemins complex, Gemins2-4, and markedly reduces the RNA and protein levels of the pro-oxydant thioredoxin-interacting protein (TXNIP) encoded by an mRNA target of Gemin5. We further show that SMN deficiency causes a dissociation of the localization of the SMN complex components from the same nuclear bodies. The accumulation of TDP-43 in SMN-positive nuclear bodies is also perturbed in SMA cells. Notably, TDP-43 is found to co-localize with SMN in nuclear bodies of flunarizine-treated SMA cells. Our findings indicate that flunarizine reverses cellular changes caused by SMN deficiency in SMA cells and further support the view of a common pathway in RNA metabolism underlying infantile and adult motor neuron diseases.

## Introduction

Spinal muscular atrophy (SMA) is an autosomal-recessive disease characterized by the degeneration of spinal cord motor neurons and muscular atrophy. It is one of the most frequent causes of infantile mortality with an estimated incidence of 1/6000–1/10000 newborns. The age of onset and a continuum of severity in SMA patients have resulted in a classification into type I (severe), type II (intermediate) and type III (moderate) forms of the disease. SMA is caused by the loss-of-function mutations of the survival motor neuron 1 (SMN1) gene leading to a deficiency in functional SMN protein (Lefebvre et al., 1995). SMN1 has a nearly identical copy, SMN2 that produces only low levels of the SMN protein (Coovert et al., 1997; Lefebvre et al., 1997). In most patients, the copy number of SMN2 parallels the severity of the disease (Melki et al., 1994; Monani, 2005; Burghes and Beattie, 2009). SMN2 is a modifier gene that partially compensates for the loss of SMN1. The C > T nucleotide difference in SMN2 leads to the exclusion of exon 7 in the majority of transcripts and to the generation of a truncated and unstable product (Lorson et al., 1999; Kashima and Manley, 2003). These results have paved the way to the emergence of innovative SMN-dependent therapies, an antisense oligonucleotide nusinersen (Rigo et al., 2012; Finkel et al., 2017; Mercuri et al., 2018) and a small-molecule risdiplam formerly RG7916 (Mercuri et al., 2019) that both modulate the inclusion of exon7 in SMN2 mRNAs and the SMN1 gene replacement AAV9-SMN1 Zolgensma (Lowes et al., 2019). These emerging therapies have improved considerably motor functions beyond time points normally reached with SMA patients. Unfortunately, the treated patients still experience substantial disability that is to varying degree to each individual (Groen et al., 2018; Singh, 2019). Therefore, there is a need to further understand how the deficiency of SMN in other cell types than motor neurons may contribute to SMA pathogenesis (Burlet et al., 1998; Hamilton and Gillingwater, 2013; Nery et al., 2019).

The evolutionary and ubiquitously expressed SMN protein is part of a large multiprotein complex formed with the Gem-associated proteins (Gemins) 2-8 and serine-threonine kinase receptor-associated protein (STRAP) also named Unrip for unr-interacting protein (Cauchi, 2010; Borg et al., 2015). The SMN complex has a general role in the assembly of RNA-proteins (RNPs) particles (Singh et al., 2017), including an essential role in the formation of the splicing small nuclear (sn)RNPs (Meister et al., 2001; Pellizzoni et al., 2002), of the small nucleolar (sno)RNPs (Jones et al., 2001; Pellizzoni et al., 2001) and of the signal recognition particles (Piazzon et al., 2013). The SMN complex assists the assembly of snRNAs (U1, U2, U4, U5, U11, U12, U4atac) with the Sm core proteins in the cytoplasm. The pre-snRNPs are then imported in the nucleus and transiently localize in nuclear bodies, specifically, Cajal bodies (CBs) for final assembly into functional snRNPs (Carvalho et al., 1999; Sleeman et al., 2001; Tucker et al., 2001; Will and Lührmann, 2001). Reduced SMN protein levels alter the snRNP repertoire in SMA patient cells and mouse models. Indeed, a reduction of the minor snRNAs (U11, U12, U4atac, and U6atac) is detected (Wan et al., 2005; Gabanella et al., 2007; Zhang et al., 2008; Lotti et al., 2012; Sapaly et al., 2018). Although widespread splicing alterations have been observed in tissues of SMA mice, the correction of the expression of Stasimon, encoded by a gene possessing a minor-intron, markedly improves motor circuit function in disease models (Imlach et al., 2012; Lotti et al., 2012). Other SMA mouse studies focusing on pre-symptomatic and in early stages of the disease indicate that a selective perturbation of the minor-intron genes may take place only in the late-symptomatic period (Bäumer et al., 2009; Zhang et al., 2013; Doktor et al., 2017). It was also reported that retained introns could promote DNA damage through the formation of RNA:DNA hybrid leading to cell death in a severe SMA mouse model (Jangi et al., 2017). It is interesting to note that SMN deficiency in lymphoblasts derived from SMA patients inhibits the splicing events mediated by the minor spliceosome (Boulisfane et al., 2011). Besides its role in snRNP biology and pre-mRNA splicing, SMN and Gemins complexes localize in dendrites and axons regulating axonal transport and local translation of specific mRNAs including those encoding for β-actin, Gap43, Anxa2, and Nrn1 proteins (Jablonka et al., 2006; Akten et al., 2011; Fallini et al., 2016; Rihan et al., 2017; Khalil et al., 2018).

The SMN protein localizes in the cytoplasm and nucleus, where it concentrates in nuclear structures called Gemini of Cajal bodies (Gems) that are found to overlap with CBs (Liu and Dreyfuss, 1996). The precise role of SMN in these nuclear structures remains unknown (Machyna et al., 2013; Sawyer et al., 2016; Staněk, 2017). Loss of SMN-positive nuclear bodies is a hallmark in SMA (Lefebvre et al., 1997; Frugier et al., 2000; Morris, 2008). They are also lost in cells of patients affected with amyotrophic lateral sclerosis (ALS), an adult-onset motor neuron disease (Yamazaki et al., 2012; Tsuiji et al., 2013; Cauchi, 2014; Sun et al., 2015; Yu et al., 2015) and in tissues of ALS mouse models carrying mutated forms of SOD1, FUS, or TDP-43 (Shan et al., 2010; Gertz et al., 2012; Kariya et al., 2012; Ishihara et al., 2013; Sun et al., 2015). Accordingly, neuronal overexpression of SMN reduces motor neurodegeneration and extended life span in mutant TDP-43 mice (Perera et al., 2016). Altogether, these observations indicate that the loss of SMN activity is associated with common disease pathways in SMA and ALS pathogenesis (Nussbacher et al., 2019). However, the exact link between spinal cord motor neurons where clinical symptoms manifest and nuclear bodies remains elusive.

To address this at the cellular level, we previously used SMA patient fibroblast cells to screen small chemical libraries for hits that could replenish nuclear Cajal bodies with SMN protein (Lefebvre et al., 2014). Flunarizine is one of the hits selected for *in vivo* studies (Sapaly et al., 2018). Interestingly, no increases of SMN protein levels were found in flunarizine-treated patient cells. We showed that flunarizine facilitates the localization of SMN in nuclear bodies, specifically, Cajal bodies of spinal motor neurons, modulates the relative abundance of specific spliceosomal snRNAs in a tissue-dependent manner and alleviates motor neuron degeneration in SMA mice. In addition, muscle atrophy and life span of SMA mouse mutants were also improved with flunarizine. However, the molecular mechanisms underlying these effects remain to be determined in cell-autonomous systems. Here, we report an increased expression of a subset of Gemins in SMA patient fibroblast cells treated with flunarizine. The molecule reduces the relative expression of the pro-oxydant TXNIP both at the RNA and protein levels, validating our RNA-seq data set. Furthermore, we show an increased localization of TDP-43 in SMN-positive nuclear bodies of flunarizine-treated SMA cells, adding to the notion of shared mechanisms in motor neuron diseases. Our results support the view that an increase in the relative protein levels of Gemins independently of the SMN protein may have beneficial outcomes in SMA.

## Materials and Methods

### Cell Culture

The immortalized Type I SMA fibroblast cell line was grown in Dulbecco’s modified Eagle’s medium (DMEM)-Glutamax supplemented with 10% fetal bovine serum (FBS), penicillin (100 U/ml) and streptomycin (100 mg/ml) at 37°C with 5% CO2 (Lefebvre et al., 2002). The flunarizine treatment was carried out as previously described (Sapaly et al., 2018). Briefly, cells are grown at a density of 10 000 cells/cm^2^ and treated with flunarizine or control DMSO (0.1%) for either 4 or 16 h.

### RNA Preparation and Expression Analysis

Total RNA was extracted with Trizol Reagent (Invitrogen Ambion) and treated with a RQ1 RNase-free DNase (Promega). One μg of RNA was used to generate cDNA with miScript II RT kit (Qiagen). Quantitative real-time PCR was performed in triplicate using SYBR Green ROX mix (Thermo Scientific) on an Applied Biosystems 7900HT. The normalized expression levels were calculated according to the ΔΔCt method. The snRNA, 5 S, 5.8 S, RPL13a and SDHA primers have been reported (Sapaly et al., 2018). The RNA sequencing approach was carried out at the Genom’ic Core Facility at the Institut Cochin, University of Paris Descartes. Briefly, ≈1 μg of total RNA with RNA Integrity Number > 8 (Bioanalyzer RNA nano chip, Agilent) isolated from cells treated with flunarizine (*n* = 3) and DMSO (*n* = 1) was used for rRNA depletion with the low Input RiboMinus Eukaryote System v2 (Ambion, Life technologies). The depleted RNAs were used to generate cDNA libraries according to the manufacturer’s protocol (Ion total RNA-Seq kit V2, Thermo Fisher Scientific). The sequencing was performed on Ion Chef (Life technologies). After quality control of the run and adaptor trimming, the reads were mapped to a reference genome using the STAR aligner. Differentially expressed genes and transcript levels were determined with the DESeq2 algorithm.

### Protein Gel Electrophoresis and Immunoblotting Experiments

The cellular extracts were prepared from fresh culture cells or frozen pellets at −80°C. The pellets were resuspended in Tris-NaCl buffer [50 mM Tris–HCl (pH 7.4), 150 mM NaCl, EDTA-free protease inhibitors cocktail (Roche)] for a Bradford protein assay (Bio-Rad). The proteins were diluted in 4X Laemmli sample buffer, resolved on 10% ProSieve polyacrylamide gel (FMC Bioproducts, Rockland, ME, United States) in Tris-Tricine running buffer, and liquid-transferred to PVDF membrane (Millipore). The immunoblots were incubated with primary antibodies diluted in 5% non-fat milk in PBS-0,5% Tween (PBS-T) for overnight at 4°C and after several washes in PBS-T, proceed for a 45-minutes incubation with HRP-conjugated secondary antibodies (1:10 000 to 1:50 000) and detected using chemiluminescence (ECL, GE Healthcare). The membranes were stripped (Restore WB stripping buffer, Thermo Scientific) and sequentially probed with antibodies. We have previously established the robustness of our experimental approach to compare the relative ECL signals (Renvoisé et al., 2012). The dilution of the antibodies was as follows: anti-SMN (BD Transduction Laboratories, Cat #610647, RRID:AB_397973, 1:1000; #502, 1:1000, Sapaly et al., 2018), anti-Gemin2 (abcam, Cat #6084, RRID:AB_305289, 1:400), anti-Gemin3 (BD Transduction Laboratories, Cat #612152, RRID:AB_399523, 1:1000), anti-Gemin4 (Santa Cruz Biotechnology, Cat #21437, RRID:AB_2111828, 1:2000), anti-Gemin5 (Sigma-Aldrich, Cat #HPA037393, RRID:AB_10672489, 1:1000), anti-Gemin8 (Sigma-Aldrich, Cat #HPA028613, RRID:AB_10602905, 1:1000), anti-unrip (abcam, Cat #102001, RRID:AB_10711545, 1:2000), anti-Y12 (abcam, Cat #3138, RRID:AB_303543, 1:2000), anti-U1-70K (ARP40276, RRID:AB_2193699, 1:1000), anti-TOE1 (Bethyl Laboratories, Cat #A303-643A, RRID:AB_11203358, 1:2000), anti-FUS (abcam, Cat #70381, RRID:AB_1271242, 1:1000), anti-TDP43 (Sigma-Aldrich, Cat #HPA017284, RRID:AB_1857775, 1:1000), anti-hnRNP A1 (Santa Cruz Biotechnology, Cat #10029, RRID:AB_648317, 1:2000), anti-hnRNP K/J (Santa Cruz Biotechnology, Cat #32307, RRID:AB_627735, 1:10 000) anti-TXNIP (NBP1-54578, RRID:AB_11033580, 1:1000), anti-α-tubulin (Sigma-Aldrich, Cat #T5168, RRID:AB_477579, 1:10000), sheep ECL anti-mouse IgG - Horseradish Peroxidase (HRP) linked species-specific whole antibody (Sigma-Aldrich, Cat #NA 931, RRID:AB_772210, 1:25000-50000), donkey ECL anti-rabbit IgG - HRP linked whole antibody (Sigma-Aldrich, Cat #NA 934, RRID:AB_772206, 1:25000-50000), and donkey anti-goat IgG – HRP secondary antibody (Santa Cruz Biotechnology, Cat #2056, RRID:AB_631730).

### Immunofluorescence Experiments

The cells grown on culture-slides or 60 mm TPP petri dishes were washed with PBS and fixed with 4% formaldehyde (FA, Sigma F-8775) in PBS, permeabilized with 0.5% Triton X-100 and immune-stained as previously described (Renvoisé et al., 2009). The dilution of the antibodies was as follows: anti-SMN (BD Transduction Laboratories, Cat #610647, RRID:AB_397973, 1:200; 4B3, 1:200, Burlet et al., 1998), anti-Gemin2 (abcam, Cat #6084, RRID:AB_305289, 1:100), anti-Gemin3 (BD Transduction Laboratories, Cat #612152, RRID:AB_399523, 1:200), anti-Gemin5 (Sigma-Aldrich, Cat #HPA037393, RRID:AB_10672489, 1:200) and anti-TDP43 (Sigma-Aldrich, Cat #HPA017284, RRID:AB_1857775, 1:200). Secondary antibodies coupled to Alexa Fluor 488 (Molecular Probes, RRID:AB_2534069, 1:400) or Cy3 (Jackson Laboratories, RRID:AB_2338254, 1:400) were used. Then DNA stained with bisBenzimide H33258 (Sigma-Aldrich Cat #B1155) and mounted in Vectashield mounting medium.

### Microscope Image Acquisition and Processing

Cells were imaged using laser-scanning confocal microscope imaging system (LSM-880, ZEISS) with a 63 × oil-immersion objective. The figures were prepared using ImageJ.

### Statistical Analyses

Analyses were performed with Excel or GraphPad software. The *P* values ≤ 0.05 were regarded as statistically significant.

### RNA Sequencing Data Sets

The files were deposited in the NCBI gene expression omnibus repository under accession number GSE145146.

## Results

### SMN Protein Is Essential for the Stability of the Gemin Components of the SMN Complexes

**FIGURE 1:**
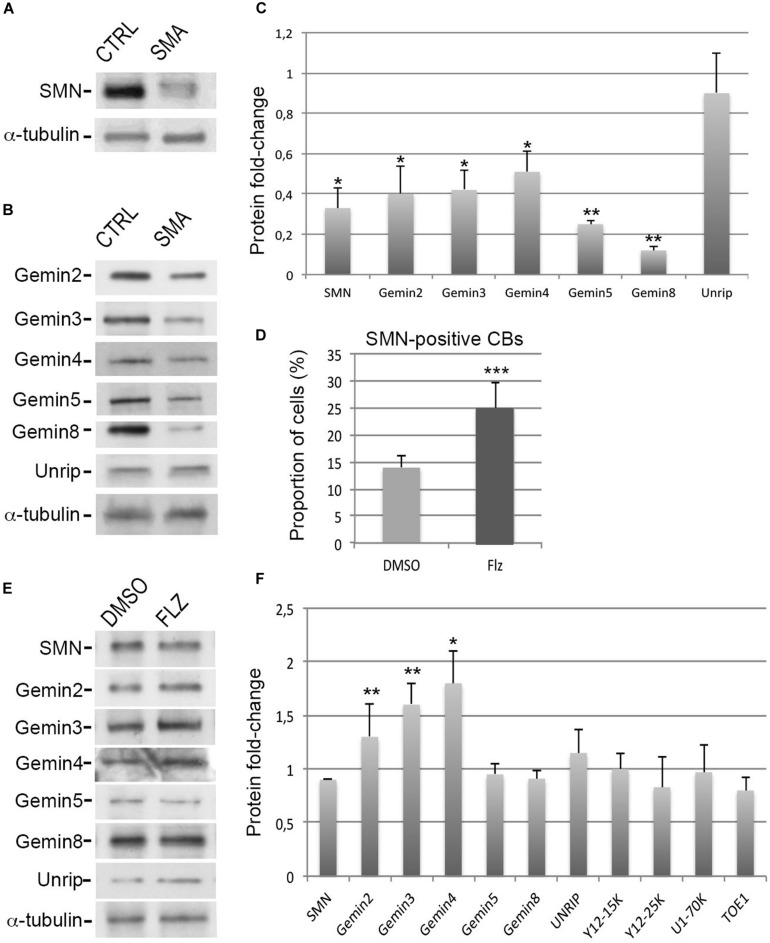
Effects of flunarizine on the protein levels of components of the SMN complex in SMA patient cells. **(A,B)** Relative protein levels of SMN and Gemins in total cell extracts of SMA patient cells compared to cells from a control individual. Proteins were resolved by SDS-PAGE and analyzed by immunoblotting using specific antibodies. **(C)** Columns represent the ratio of each protein over α-tubulin that served as the loading control and are normalized to the ratios of control fibroblasts (Arbitrary unit of 1). Errors bars indicate the S.E.M. (3 independent experiments, Student’s *t*-test, **P < 0.01, *P < 0.05). **(D)** Statistical analysis of SMN positive-nuclear Cajal bodies. (>300 cells, *n* = 3 independent experiments). Error bars indicate the standard deviation (S.D.), ***P < 0.001 (Khi-2 test). **(E)** Protein expression of SMN complex components in total cellular extracts from SMA patient cells following flunarizine treatment compared to control DMSO treatment. **(F)** Columns represent the ratio of each protein over α-tubulin that served as the loading control, and are normalized to the ratios obtained following the control DMSO treatment (Arbitrary unit of 1). (3 ≤ *n* ≤ 6 independent experiments, Student’s *t*-test, **P < 0.01, *P < 0.05).

An immortalized type I SMA patient-derived fibroblast cell line (from here on simply referred to as SMA cells) was previously established (Lefebvre et al., 2002). Given that the expression levels of the SMN complex components are inter-dependent (Feng et al., 2005; Gabanella et al., 2007), we performed immunoblot experiments with antibodies against Gemins and unrip components to analyze the protein levels in these SMA cells (Figures 1A–C). Compared to controls, a significant 50–70% reduction of SMN, Gemin2, Gemin3 and Gemin4 was observed whereas unrip was not significantly reduced. Gemin5 and Gemin8 were the most affected by low levels of SMN with a significant 80–90% reduction. Such a reduction of Gemin5 was not previously reported (Gabanella et al., 2007). Our data agree with our prior observation that the production of the SMN complex is less efficient in SMA cells compared to controls (Renvoisé et al., 2012). Our results are also in agreement with past studies that estimate SMN protein levels to be reduced by 50–75% that of controls in SMA patient fibroblasts (Sumner et al., 2003). Consistent with a 50–70% reduction of *in vitro* snRNP assembly activity in SMA fibroblast cell extracts (Wan et al., 2005; Gabanella et al., 2007), an overall reduction of the snRNA levels was also noted here in SMA cells compared to controls (Supplementary Figure S1C). Our observations indicate that the degrees of protein reduction differ among the components of the SMN complex in SMA cells.

### Flunarizine Modulates the Protein Levels of SMN Complex Components in SMA Patient Cells

To investigate a potential link between flunarizine and the SMN complex, we asked whether the molecule could modulate the protein levels of the components of the complex in SMA cells. First, we evaluated the effects on the number of SMA cells with SMN-positive Cajal bodies after 4-hours incubation with flunarizine or control DMSO (Figure 1D). Flunarizine led to a significant increase in the proportion of cells with more SMN nuclear Cajal bodies than DMSO-treated cells (khi-2 test, *P* < 0.001). These data indicate that flunarizine recruits SMN to Cajal bodies as early as 4-hour after the administration of the molecule. Then, total cell extracts were prepared from flunarizine and DMSO-treated SMA cells and proteins were analyzed by immunoblotting (Figures 1E,F). Immunoblots revealed that Gemin2, 3 and Gemin4 accumulated to higher protein levels with flunarizine whereas SMN, Gemin5, Gemin8 and unrip levels were not changed, indicating a separation of the SMN-complex components by flunarizine in SMA cells. The SMN complex assembles the snRNP core Sm proteins on the snRNAs (Battle et al., 2006), therefore we also tested the protein level of the snRNP core Sm proteins using anti-Y12 (recognizing SmB/B’), of a specific U1 snRNP protein using anti-U1-70K and of a factor associated with snRNP homeostasis using anti-target of EGR1 protein 1 (TOE1, Fong et al., 2013) antibodies (Supplementary Figures S1A,B). No differences were detected with these snRNP-associated or related proteins. It is consistent with the homeostasis of the snRNP subunits being preserved through a safeguarding system (Prusty et al., 2017). Our data indicate that flunarizine does not affect the steady-state levels of proteins implicated in the snRNP biology other than Gemins in SMA cells.

### Flunarizine Does Not Change the Global Levels of Splicing snRNA in SMA Patient Cells

**FIGURE 2:**
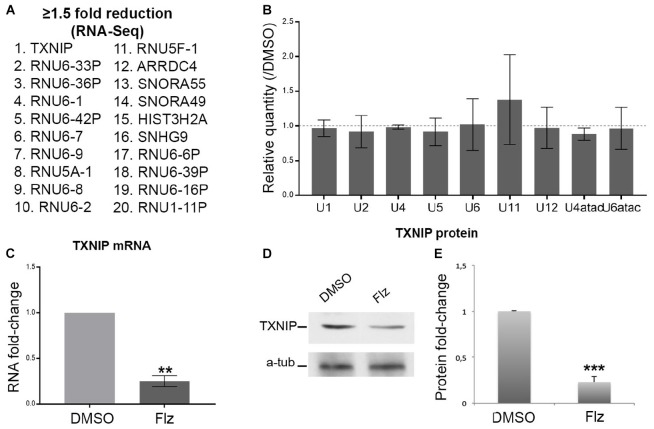
Effects of flunarizine on the expression of target RNAs in SMA patient cells. **(A)** The twenty most reduced genes identified by RNA sequencing after 4-hour of flunarizine treatment are listed. **(B)** The snRNA levels are determined by RT-qPCR and the relative amount is presented as fold-change of the flunarizine (Flz) treatment compared to DMSO (arbitrary unit of 1). The 5 S and 5.8 S are used as controls for normalization as described previously (Sapaly et al., 2018). Error bars indicate the S.E.M (five independent experiments, Student’s *t*-test, not significant). **(C)** Quantitative RT-PCR analysis confirms that flunarizine reduces TXNIP mRNA expression in SMA cells. RPL13 and SDHA are used as internal controls for normalization (Three independent experiments, Student’s *t*-test, **P < 0.01). **(D)** Immunoblot analysis using specific antibodies shows that flunarizine reduces the protein levels of TXNIP. **(E)** Columns represent the ratio of the protein over α-tubulin that served as the loading control and normalized to the ratios obtained following the DMSO treatment (arbitrary unit of 1) (four independent experiments, Student’s *t*-test, ***P < 0.001).

To gain insights into the molecular events associated with the drug, a genome-wide RNA-sequencing approach was performed to determine gene expression (to be published in details elsewhere). Several snRNA U5 and U6 variants and small nucleolar (sno) RNAs were reduced by flunarizine as listed in Figure 2A. The most significantly reduced mRNAs were coding for thioredoxin-interacting protein (TXNIP) and arrestin domain containing 4 (ARRDC4) proteins, two members of the alpha-arrestin protein family that have been shown to regulate cellular metabolism (Patwari et al., 2009). Interestingly, Gemin5 regulates internal ribosome entry sites in the 5′ untranslated region of TXNIP mRNA (Lampe et al., 2018). Given that flunarizine affects *in vivo* the relative snRNA levels in brain and spinal cord of SMA mice (Sapaly et al., 2018), we hypothesized that even small changes in protein levels of the SMN complex components can lead to larger effects on their target RNAs. Therefore, we evaluated by RT-qPCR the levels of the spliceosomal snRNAs in total RNA preparations. No significant differences in snRNA levels were observed from flunarizine- and DMSO-treated SMA cells (Figure 2B). These results contrast with the analyses in SMA mice treated with flunarizine (Sapaly et al., 2018). However, it agrees with a more rapid turn over of the snRNAs (Fury and Zieve, 1996; Younis et al., 2013; Venters et al., 2019) and with the view that unstable snRNA pools outside ribonucleoprotein particles might be rather small (Supplementary Figure S1C; Sauterer et al., 1988; Zieve et al., 1988; Zhang et al., 2008). Unfortunately, the low levels of expression and the sequence identity of the different snRNAs found in our RNA-Seq data set hampered specific RT-qPCR analyses. Moreover, considering that flunarizine reduces the mRNA levels of TXNIP, a cellular redox regulator, in HeLa cells (Younis et al., 2010) and in the brains of SMA mice (Sapaly et al., 2018), we examined its expression both at the mRNA and protein levels in treated SMA cells. Txnip mRNA levels were significantly reduced by ≈4-fold with flunarizine compared to the DMSO treatment and was confirmed by a significant ≈3-fold reduction at the protein levels (Figures 2C,D). Altogether, these data validate the results of the RNA sequencing approach after 4 hours of flunarizine treatment of the SMA cells.

### Flunarizine Influences the Nuclear Localization of SMN-Complex Components in SMA Patient Cells

**FIGURE 3:**
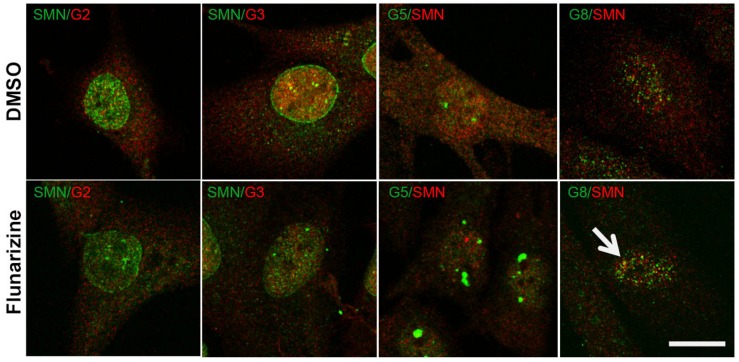
Sub-cellular localization of SMN with Gemins 2, 3, 5, and 8 following flunarizine treatment. Fixed SMA cells are analyzed under confocal microscopy for the presence of Gemins in SMN-positive nuclear bodies. Immunostaining of SMN with rabbit polyclonal antibodies (in green) is used with mouse monoclonal antibodies against Gemins 2 and 3 (in red). The mouse monoclonal antibodies against SMN (in red) are used with rabbit antibodies against Gemin 5 and Gemin8 antibodies (in green). The co-localization of proteins with SMN results in a yellow signal (arrow). Scale bar: 10 μm.

**FIGURE 4:**
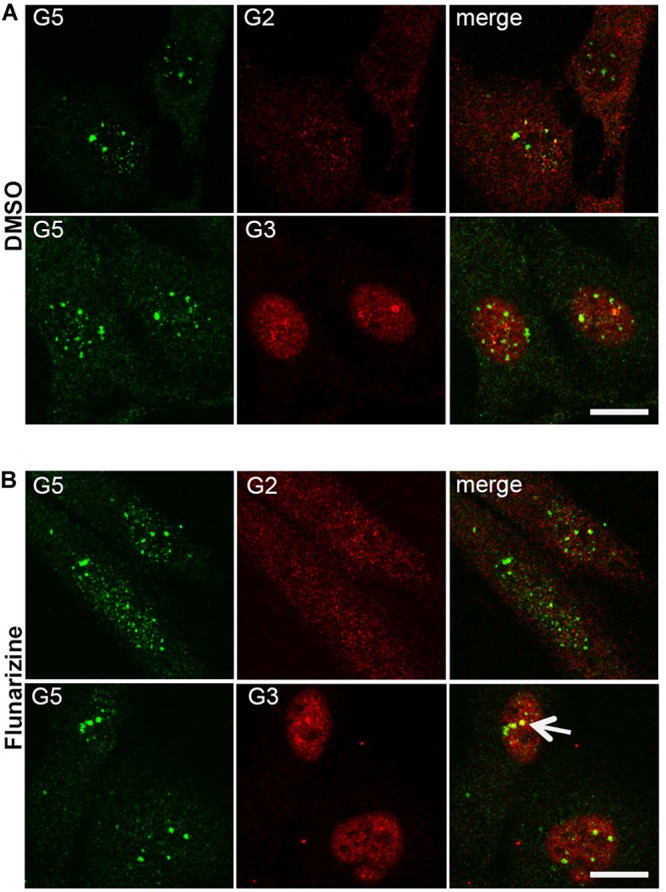
Sub-cellular localization of Gemin5 with Gemins 2 and 3 following flunarizine treatment. Fixed SMA cells were analyzed under confocal microscopy for the presence of Gemin2 or 3 in Gemin5-positive nuclear bodies. Immunostaining of Gemin5 with rabbit polyclonal antibodies (in green) is used with mouse monoclonal antibodies against Gemins 2 and 3 (in red). The co-localization of proteins with Gemin5 results in a yellow signal (arrow). Scale bar: 10 μm.

Given that flunarizine influences the formation of SMN-positive nuclear bodies both *in vitro* and *in vivo* (Sapaly et al., 2018), confocal microscopy was used to evaluate the subnuclear localization of some SMN complex components in flunarizine-treated SMA cells. The SMN complex is found in both the cytoplasm and the nucleus, where it concentrates in either gems and/or CBs. We performed immunofluorescence double labeling for the SMN and Gemins (Figure 3). SMN protein poorly co-localized or was found adjacent to Gemin2, Gemin3, Gemin5 or Gemin8 in the nucleus of SMA cells. Flunarizine treatment did not enrich the Gemins in SMN-positive bodies of SMA cells, the most co-localized being Gemin8. The most striking observation was the accumulation of Gemin5 in large nuclear bodies. Previous protein-protein interaction studies established that Gemin5 interacts directly with SMN/Gemin2 sub-complex (Gubitz et al., 2002; Otter et al., 2007) and could be found in discrete complex with SMN and Gemin3 upon translation inhibition (Yong et al., 2010). We then tested whether Gemin5 was co-localized with Gemin2 or Gemin3 (Figure 4). Double immunolabelling showed that in 50% of the flunarizine-treated SMA cells Gemin3 accumulated with Gemin5-positive nuclear bodies when compared with 35% of DMSO-treated cells (698 cells, 3 independent experiments, khi-2, *P* < 0,001) whereas Gemin2 did not significantly accumulate within Gemin5-positive nuclear bodies with the drug (627 cells, 3 independent experiments, 0,1 > *P* > 0,05). These results indicate that the SMN complex components are only weakly associated to the same nuclear structures when the SMN protein levels are reduced in SMA cells.

### Flunarizine Modulates the Levels of RNA-Binding Proteins Relevant to SMA and ALS Motor Neuron Diseases

**FIGURE 5:**
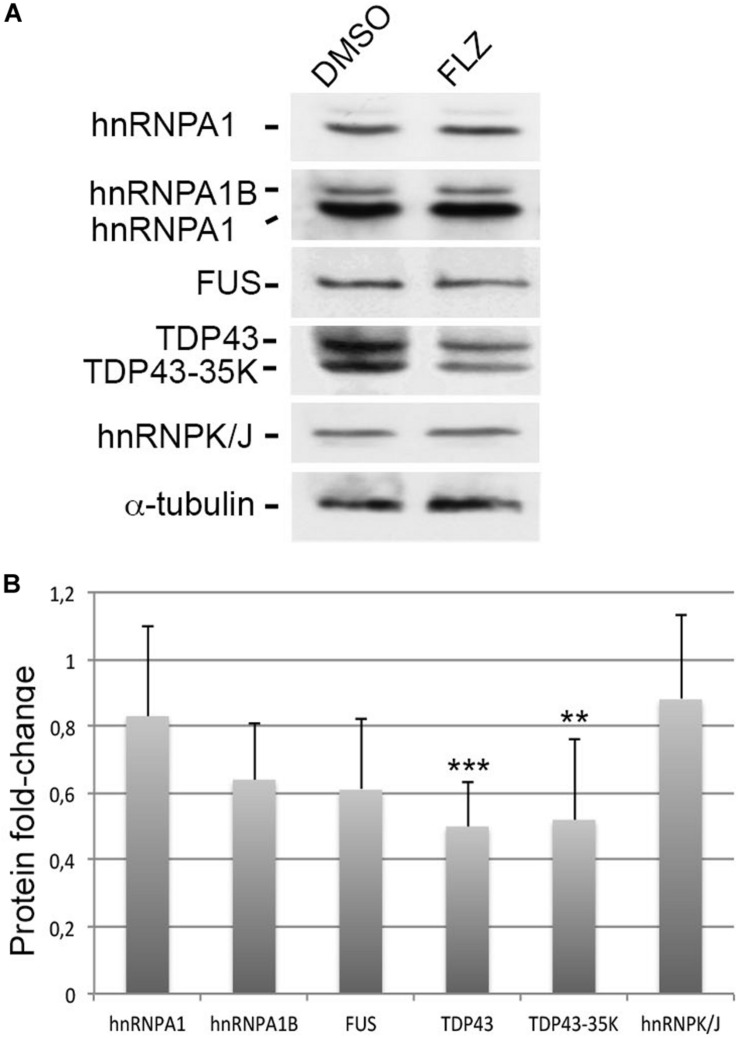
Effects of flunarizine on the protein levels of RNA-binding proteins relevant to motor neuron diseases in SMA patient cells. **(A)** Protein expression of hnRNPA1, FUS and TDP-43 in total cellular extracts from SMA patient cells following flunarizine treatment compared to control DMSO. Proteins were resolved by SDS-PAGE and analyzed by immunoblotting using specific antibodies. The α-tubulin served as the loading control. **(B)** Columns represent the ratio of each protein over α-tubulin that served as the loading control, and normalized to the ratios obtained following the control DMSO treatment (arbitrary unit of 1) (three independent experiments, Student’s *t*-test, ***P < 0.001, **P < 0.01).

Given that RNA-binding proteins such as hnRNP A1, FUS, and TDP43 have been associated with snRNP biology and splicing defects in ALS motor neuron diseases (Nussbacher et al., 2019), we asked whether they could be modulated by flunarizine at the protein level (Figure 5). The hnRNP A1 proteins contribute to the disease severity in SMA and ALS motor neuron diseases (Bekenstein and Soreq, 2013). In addition, the hnRNP A1B isoform is regulated by TDP-43 and is proned to aggregation in ALS patient motor neurons (Deshaies et al., 2018). Our immunoblots revealed that the protein levels of the canonical hnRNP A1 and A1B isoforms, and of FUS were not significantly reduced upon flunarizine treatment of SMA cells compared with DMSO-treated cells (three independent experiments, Student’s *t*-test). The analysis of the immunoblots performed with anti-TDP-43 antibodies revealed a significant 50% reduction of TDP-43 protein levels in flunarizine-treated SMA cells compared to the DMSO treatment (three independent experiments, Student’s *t*-test, *P* = 0.005). Also, the incubation with the anti-TDP-43 antibodies revealed a protein migrating at 35K that was also significantly modulated by the drug in SMA cells (Student’s *t*-test, *P* = 0.01). These results indicate that the levels of RNA-binding proteins could be influenced by flunarizine in SMA cells.

### Protein Levels and Localization of TDP-43 Are Markedly Altered in SMA Patient Cells

**FIGURE 6:**
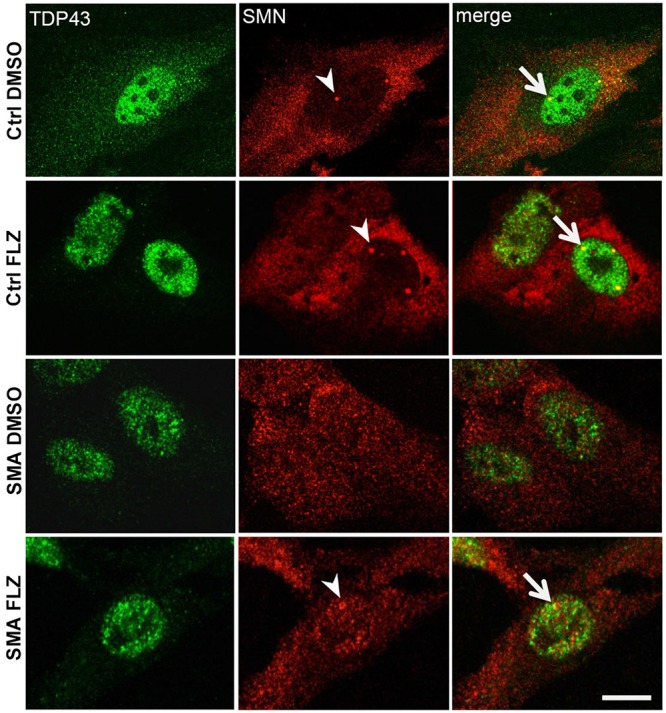
TDP-43 nuclear bodies are reduced in SMA patient cells. Fixed control and SMA cells were analyzed under confocal microscopy for the presence of TDP-43 in SMN-positive nuclear bodies upon flunarizine treatment. Cells from a control individual treated with DMSO (ctrl DMSO) reveal a nuclear and cytoplasmic labeling for TDP-43 whereas the cytoplasmic labeling reduces upon flunarizine treatment (ctrl FLZ). TDP-43 protein is found co-localized with SMN (arrowhead) in nuclear bodies (arrows) as shown in yellow in flunarizine-treated control and SMA cells. Scale bar: 10 μm.

Previous studies showed a marked reduction of components in Cajal bodies of SMA patient cells (Renvoisé et al., 2006, 2009). SMN protein levels are markedly reduced in SMA cells (Figure 1A) that could also alter the nucleocytoplasmic distribution of RNA-binding proteins such as TDP-43. We examined here by immunofluorescence experiments the localization of SMN and TDP-43 proteins in DMSO and flunarizine-treated control and SMA cells. In control cells, SMN was distributed in the cytoplasm and concentrated in nuclear bodies whereas it was found in the nucleoplasm of SMA cells with altered immune-labeling signals (Figure 6). In control cells, TDP-43 was predominantly in the nucleus and co-localized with SMN in prominent nuclear bodies. The nuclear staining of TDP-43 was stimulated by flunarizine whereas its cytoplasmic staining disappeared in control cells. Although markedly reduced in SMA cells, the TDP-43 staining was almost exclusively nuclear. Double immunolabelling showed that in 20% of the flunarizine-treated SMA cells TDP-43 accumulated with SMN-positive nuclear bodies when compared with 5% of DMSO-treated cells (627 cells, 3 independent experiments, khi-2 test 0.02 > *P* > 0.01), indicating that flunarizine induced a 4-fold increase of the co-localization of SMN and TDP-43 in nuclear bodies of SMA cells. These results suggest that the localization of TDP-43 in nuclear bodies is dependent on the presence of SMN that is induced by flunarizine.

## Discussion

The deficiency of the ubiquitous SMN protein causes SMA. However, the pathogenesis of SMA is not fully understood. The SMN-Gemins complexes concentrate in nuclear-body Cajal bodies that are lost in SMA and markedly reduced in ALS patient cells. Flunarizine increases SMN in nuclear Cajal bodies of motor neurons, extends life span and attenuates motor neuron degeneration in SMA mutant mice. However, the precise mode of action remains to be determined. We analyzed here the impact of flunarizine using an immortalized fibroblast cell line derived from a severe Type I SMA patient, taking advantage of the genetic context of the human disease. We found that SMN deficiency is associated with low levels of Gemins in SMA patient cells. We showed that flunarizine diminishes the deficiency of Gemins 2, 3 and 4. We also showed that SMN and TDP-43, a protein implicated in ALS disease, are both recruited to the same nuclear bodies with flunarizine.

An additional functional link between SMN and TDP-43 comes from our findings in cells treated with flunarizine (Figure 6). We show that SMN protein in cells from a control individual promotes the accumulation of TDP-43 in SMN-positive nuclear bodies as previously reported in neurons (Tsuiji et al., 2013) that is enhanced here with flunarizine. We also show that the immunostaining of TDP-43 is markedly reduced in SMA patient cells. This is in agreement with an early reduction of RNA-binding proteins with the age of onset of the clinical symptoms in motor neuron diseases. Indeed, SMA is probably more severe that ALS-associated TDP-43 disease since both proteins are reduced earlier in the life of patients. The reverse might also be true because the overexpression of SMN in a TDP-43 mouse model ameliorates the pathological signs (Perera et al., 2016). Our observations further suggest that both proteins are linked to some aspects of RNA metabolism perturbed in common amongst early onset motor neuron diseases.

TDP-43 is a nuclear protein involved in splicing regulation and mRNA stability (Lagier-Tourenne et al., 2010). It is also found in the cytoplasm that increases upon stress induction (Li et al., 2013). This is normally a reversible process. Indeed, we show here that flunarizine inhibits the cytoplasmic localization of TDP-43 in fibroblasts from a control individual, further indicating that its cytoplasmic distribution can be reversed independently of its nuclear counterpart. In ALS conditions, the ALS-linked TDP-43 mutations cause constant large cytoplasmic aggregates of the mutant proteins conferring some toxicity (Johnson et al., 2009). On the other hand, the nuclear depletion of TDP-43 decreases gene expression and alters the stress granules homeostasis. Indeed, normal cytoplasmic stress granules may have a cytoprotective role in neurodegeneration (Wolozin, 2012). TDP-43 regulates stress granules in cells expose to oxidative stress (Dewey et al., 2011). These granules facilitate cellular survival through the storage of mRNAs and RNA-binding proteins during stress conditions. Other studies have shown that oxidative stress relocalizes Gemin5 to stress granules but not SMN and Gemin2 (Battle et al., 2007). Moreover, during stress exposure, mRNAs are often translated using non-canonical translation mechanisms (Spriggs et al., 2008). Those mRNAs contain internal ribosomal entry sites (IRES) that allow for cellular *trans-*acting factors to recruit the translation machinery in a cap-independent manner (Kwan and Thompson, 2019). A novel IRES has been found in the 5′UTR of the TXNIP mRNA that is regulated by factors including Gemin5 (Lampe et al., 2018). Interestingly, flunarizine reduces here the pro-oxydant TXNIP both at the mRNA and protein levels (Figure 2). TXNIP binds and inhibits the reductase activity of the thioredoxin, an important cellular system against oxidative stress (Spindel et al., 2012). Therefore, flunarizine should improve the intracellular redox status in SMA conditions.

Previous immunohistochemical analyses of CB components revealed that only a subset of functions is impaired in SMA patient cells (Renvoisé et al., 2006, 2009). Here we have extended the list of proteins tested to the Gemins. We have found that Gemin5 resides in prominent nuclear bodies separated from the other components of the SMN complex. This is probably the consequence of the low protein levels of SMN and Gemins expressed in these cells. Gemin5 is the RNA-binding protein with multi-functional domains (Piñeiro et al., 2015). It delivers the pre-snRNA to the SMN complex for the U snRNP assembly (Battle et al., 2006; Otter et al., 2007). Multiple interactions are taking place to form a functional SMN complex. SMN binds directly to Gemin2, Gemin3 and Gemin8. Gemin5 binds to SMN-Gemin2 subunit. Gemin 3 and Gemin8 bind to Gemin4. Finally, Gemin8 recruits the Gemin6-Gemin7-unrip to the complex. Moreover, several Gemins are also found in complexes outside the SMN complex. To note, Gemin5 is found associated with Gemin3 and Gemin4 in a SMN-free complex (Battle et al., 2006). It is interesting to note that flunarizine increases the protein levels of Gemins interacting directly with Gemin5, namely Gemins 2-4 (Figure 1) and recruits Gemin3 in Gemin5-positive nuclear bodies (Figure 4). Therefore, it is tempting to speculate that Gemin5 and/or its interactors are direct target(s) of flunarizine. We also show that flunarizine decreases the levels of another RNA-binding protein (Figure 5). SMN complex associates with a number of various RNA-proteins particles (Singh et al., 2017). Indeed, it has been proposed that SMN-Gemin2 is a versatile hub for chaperoning those particles (So et al., 2016). One plausible outcome of the flunarizine treatment is that a decrease in protein levels of SMN-Gemin2 “clients” will favor its association with gemin3-4 and the U snRNP assembly. This does not exclude the possibilities that other functions separate from snRNP assembly to be influenced by flunarizine. A protein complex formed of Gemins3-5 has been found associated with microRNAs (Mourelatos et al., 2002). It will be interesting to determine in the future whether flunarizine also modulates microRNA metabolism in SMA.

Finally, we have shown here that flunarizine enhances the protein levels of Gemins independently of SMN. This offers an interesting perspective in term of combinatorial therapies against SMA. Indeed, we have previously shown that flunarizine improves the clinical signs of an SMA mouse model (Sapaly et al., 2018). Taken together our observations favor the view of a role for the Gemins in the disease phenotype (Battle et al., 2007) and being potential therapeutic targets in motor neuron diseases. Although mRNA levels for Gemin2 are not reduced in SMA patient cells (Helmken et al., 2000), we show here a marked reduction of Gemin2 at the protein level in SMN-deficient patient cells. Our results are in agreement with a previous report of mice double heterozygous deficient for SMN and Gemin2 exhibiting enhanced loss of spinal motor neurons, a hallmark of SMA disease (Jablonka et al., 2002). It is possible that the current SMN-dependent therapies of SMA might not cause sufficient long-term phenotype improvements for all patients (Singh, 2019) and might also require increasing the functions of the components of the SMN-Gemins complex. Pharmacological approaches to augment the protein levels of the Gemins may therefore be justified in combination with the recently approved SMA therapies that up-regulate SMN protein levels (Borg and Cauchi, 2014). Thus, we expect that a more detailed understanding of the molecular mechanisms of SMA disease should benefit to the development of the future therapies against motor neuron diseases.

## Data Availability Statement

The files were deposited in the NCBI gene expression omnibus repository under accession number GSE145146.

## Author Contributions

DS and PD conducted the RT-qPCR and their analyses. SL performed the cell culture, immunoblot and immunofluorescence experiments and the microscopy studies with JC. SL and BS quantified the co-localization observations. FD conducted the RNA-seq experiments. FL performed the bio-informatics analyses of the RNA-seq datasets. SL initiated, conceived, and supervised the project, analyzed the results, and wrote the manuscript. All authors commented on the manuscript.

## Conflict of Interest

The authors declare that the research was conducted in the absence of any commercial or financial relationships that could be construed as a potential conflict of interest.

## Supplementary Material

FIGURE S1 Levels of snRNP proteins in flunarizine-treated SMA patient fibroblasts compared to DMSO treatment and the relative snRNA levels in SMA cells compared to cells from a control individual. **(A,B)** Protein expression of snRNP metabolism in total cellular extracts from SMA patient cells following flunarizine treatment compared to control DMSO treatment. **(C)** The snRNA levels in SMA fibroblasts are determined by RT-qPCR and the relative amount is presented as fold-change compared to fibroblasts from a control individual (arbitrary unit of 1). The 5 S and 5.8 S are used as internal controls for normalization as described previously (Sapaly et al., 2018). Error bars indicate the S.E.M (five independent experiments, Student’s *t*-test, ^∗∗∗^*P* ≤ 0.003, ^∗∗^*P* < 0.01).
