# Supplementary figures and images for: The Small-Molecule Flunarizine in Spinal Muscular Atrophy Patient Fibroblasts Impacts on the Gemin Components of the SMN Complex and TDP43, an RNA-Binding Protein Relevant to Motor Neuron Diseases
Source: Front Mol Biosci. 2020 Apr 17;7:55. doi: 10.3389/fmolb.2020.00055 (PMC7181958; doi:10.3389/fmolb.2020.00055)

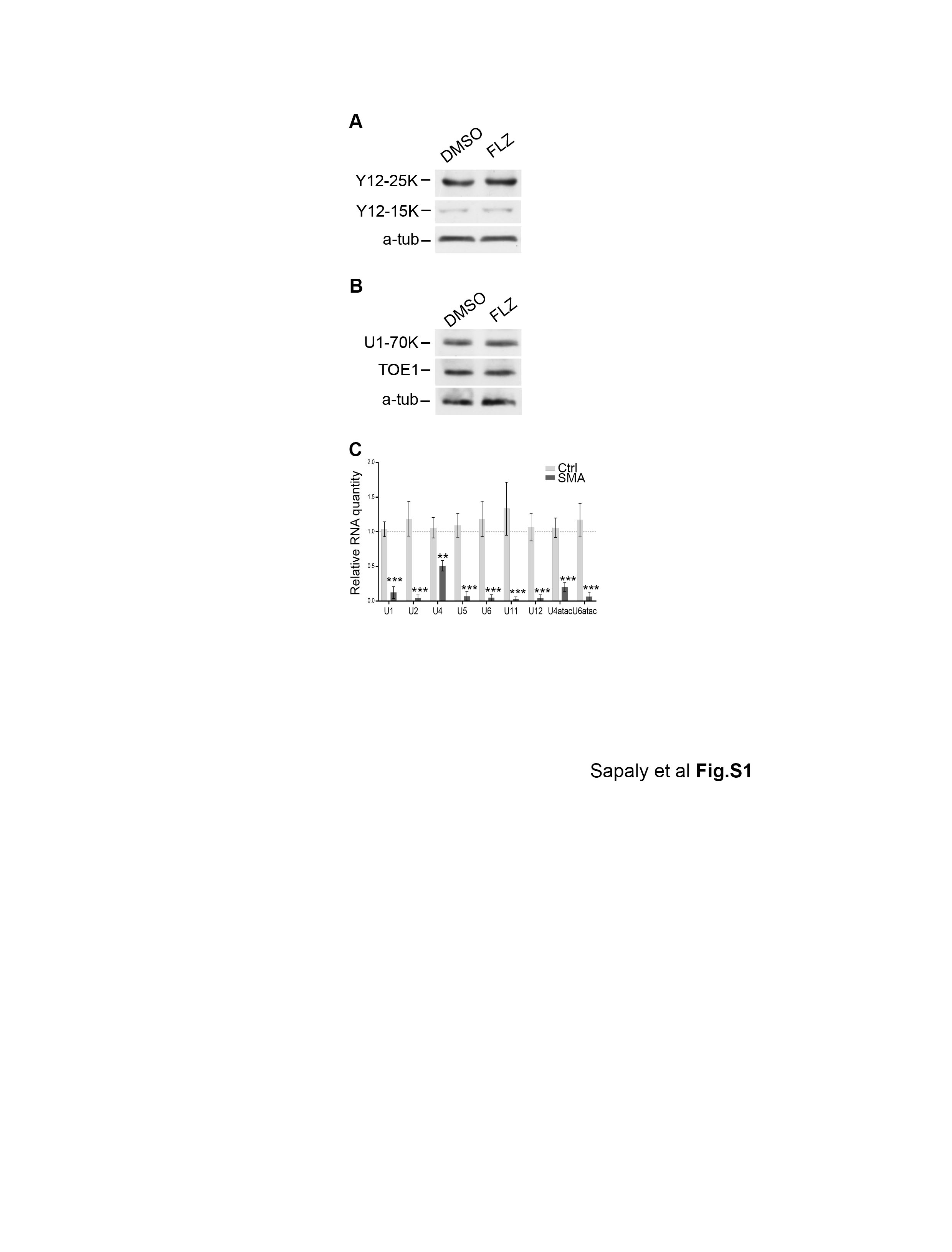

Supplement: FIGURE S1 — Levels of snRNP proteins in flunarizine-treated SMA patient fibroblasts compared to DMSO treatment and the relative snRNA levels in SMA cells compared to cells from a control individual. (A,B) Protein expression of snRNP metabolism in total cellular extracts from SMA patient cells following flunarizine treatment compared to control DMSO treatment. (C) The snRNA levels in SMA fibroblasts are determined by RT-qPCR and the relative amount is presented as fold-change compared to fibroblasts from a control individual (arbitrary unit of 1). The 5 S and 5.8 S are used as internal controls for normalization as described previously (Sapaly et al., 2018). Error bars indicate the S.E.M (five independent experiments, Student’s t-test, ∗∗∗P ≤ 0.003, ∗∗P < 0.01). [file Image_1.jpeg]
